# The impact of the COVID-19 pandemic on the rehabilitation therapy of children and adolescents with cerebral palsy: a nationwide, health insurance data-based study

**DOI:** 10.3389/fpubh.2024.1374766

**Published:** 2024-05-30

**Authors:** Jieun Shin, Mi Jin Hong, Jong Bum Park, Yung Jin Lee

**Affiliations:** ^1^Department of Biomedical Informatics, College of Medicine, Konyang University, Daejeon, Republic of Korea; ^2^Department of Rehabilitation Medicine, College of Medicine, Konyang University Hospital, Daejeon, Republic of Korea

**Keywords:** rehabilitation therapy, rehabilitation impact, cerebral palsy, children and adolescents, COVID-19

## Abstract

**Introduction:**

The coronavirus disease 2019 (COVID-19) pandemic has profoundly affected the utilization of rehabilitation services. Existing evidence investigating this issue at the nationwide level is lacking, and it is uncertain whether the effects of the COVID-19 pandemic on the use of rehabilitation therapy of children and adolescents with cerebral palsy. This study aimed to investigate the impact of COVID-19 on the rehabilitation therapy of children and adolescents with cerebral palsy.

**Methods:**

We obtained data from South Korea’s Health Insurance Review and Assessment Agency for 2017–2021. By analyzing the claims data, we focused on rehabilitation therapy in individuals with CP under 18 years of age. We categorized these according to therapy type (physical, occupational, or dysphagia), medical facility, hospital visits, and insurance. We calculated the patient counts and average claims per person and compared the average from before to during the COVID-19 pandemic.

**Results:**

Over the 5 years, there was a significant decline in the number of patients undergoing rehabilitation therapy (trend *p* = 0.004), but the average claims per person remained stable (trend *p* = 0.971). During the COVID-19 pandemic, the average number of claims per person decreased significantly compared to the control period (*p* = 0.013). Both the physical (*p* = 0.049) and occupational therapy groups (*p* = 0.019) showed significant differences in claims. General hospitals and hospitals experienced a decrease in average cases by 2.2 (*p* < 0.001) and 2.4 (*p* < 0.001) respectively, while long-term care hospitals increased by 3.1 cases (*p* < 0.001). Outpatients showed a decline of 2.0 cases (*p* < 0.001), whereas inpatients showed an increase of 5.9 cases (*p* < 0.001). Individuals with health insurance decreased by 0.5 cases (*p* = 0.007), but the decrease of 0.08 cases among medical aid-covered individuals was not statistically significant (*p* = 0.898).

**Conclusion:**

In 2020–2021, the average number of claims per person showed a significant decrease compared to the pre-COVID-19 pandemic period (2017–2019). Depending on the type of treatment, the number of claims for physical and occupational therapy significantly decreased.

## 1 Introduction

Coronavirus disease 2019 (COVID-19) has spread worldwide since December 2019 (1). On 20 January 2020, the first case of severe acute respiratory syndrome coronavirus 2 was reported in South Korea (2), and the World Health Organization declared COVID-19 a pandemic on March 11, 2020. To control COVID-19, South Korea took essential steps, such as social distancing and mandatory face masks, on 22 March 2020, to reduce the impact of the pandemic (3). Owing to social distancing, interactions between individuals and the utilization of clinical services decreased. Furthermore, unexpected social distancing impacted rehabilitation therapy, because many components of rehabilitation care require patient contact.

Cerebral palsy (CP) causes motor function impairments and is accompanied by sensory, cognitive, communicative, and nutritional problems (4). Abnormal movements and changes in posture emerge early in life, and subsequent impairments can progress. Therefore, CP management involves multiple experts and rehabilitation programs. Rehabilitation therapy for CP is individualized for each patient, with physical therapy and occupational therapy playing crucial roles. The therapy includes various methods and approaches, such as stretching, massage, strength training, weight-bearing exercises, balance activities, electrical stimulation, and endurance training. Additionally, for the treatment of patients with hemiplegia, therapies such as constraint-induced movement therapy and hand-arm intensive bimanual therapy can be implemented to improve upper limb function (5). Extended periods of reduced access to rehabilitation services for children with CP can impact functionality, mental health, and psychological well-being (6).

During the pandemic, the suspension of in-person services significantly affected children and adolescents with disabilities who relied on specialized services for therapeutic activities, social interaction, and the development of adaptive skills. For social distancing, many rehabilitation and special education centers reduced services provided to children with disabilities (7). Although studies have been conducted on the impact of the pandemic on individuals with disabilities and in the field of rehabilitation, no research has specifically examined the practical changes that have occurred.

Therefore, we aimed to investigate the effects of COVID-19 on rehabilitation therapy in children and adolescents with CP using claims data from the Korean National Health Insurance Review and Assessment Service (HIRA) before and during the pandemic to compare the number of patients and the average number of claims per person.

## 2 Materials and methods

### 2.1 Data source

This was a registry-based study of patients diagnosed with CP who underwent rehabilitation therapy in South Korea using the Korean HIRA database. This governmental entity evaluates the appropriateness of fees levied by healthcare facilities and mandates payments to the Korea Health Insurance Corporation (8). All South Koreans are required to enroll in the National Health Insurance System (NHIS), and the HIRA database provides researchers with data on the healthcare services used by the entire population (9). Additionally, because the HIRA reviews claims data for the remaining 3% of the population enrolled in the National Medical Aid program, it encompasses nearly all inpatient and outpatient records.

### 2.2 Patient selection and variables

We identified participants from the HIRA database between January 2017 and December 2021, including patients diagnosed with CP (International Classification of Diseases, 10th revision [ICD-10] code G80); ≤18 years; and initially selected from the claims database with data corresponding to rehabilitation services. Only professional rehabilitation treatments were included, defined by The National Health Insurance Corporation as rehabilitation performed by a specialist or therapist on a one-to-one basis. The rehabilitation claim codes used for tracking included MM105 (rehabilitative development therapy for disorder of the central nervous system), MM301 (mattress or mobilization training), MM302 (gait training), MM112 (complex occupational therapy), MM113 (special occupational therapy), MM114 (activities of daily living training), MX141 (rehabilitative dysphagia therapy), and MZ008 (functional electrical stimulation for rehabilitative dysphagia therapy). Claims data from Oriental medical facilities were excluded.

We categorized the claims data into three groups according to the following criteria for similar therapeutic codes: physical therapy (PT) group (MM105, MM301, MM302); occupational therapy (OT) group (MM112, MM113, MM114); and dysphagia therapy (DT) group (MX141, MZ008). The claims database includes demographic data and diagnoses of patients, types of rehabilitation therapy (such as PT, OT, and DT), types of medical institutions (including tertiary hospitals, general hospitals, hospitals, clinics, and long-term care hospitals), visits to medical institutions (including inpatient and outpatient visits), and types of insurance (e.g., national health insurance and medical aid). Based on the date of the first diagnosis of COVID-19 in South Korea (January 2020), we determined that 2017 to 2019 was the pre-pandemic period and 2020 to 2021 was the pandemic period.

Outcomes were the number of individuals who underwent rehabilitation, average claims per individual, and initial attributes. Additionally, we analyzed the average claims per individual before and after the pandemic. We analyzed yearly variations in the trends of rehabilitation prescription counts and patterns before and after the emergence of COVID-19, type of treatment, medical facilities, visits, and insurance.

### 2.3 Statistics analysis

Baseline characteristics were summarized using descriptive statistics and are presented as frequencies and percentages. We conducted a regression analysis to examine changes in claims frequency and number of patients overall and by variables from 2017 to 2021. Subsequently, an independent t-test was used to compare the average number of claims per person before and after the pandemic. The level of statistical significance for all tests was set at *α* = 0.05. All statistical analyses were performed using SPSS version 26 (IBM Corp., Armonk, NY). All statistical analyses were two-tailed, and *p* values <0.05 were considered statistically significant.

## 3 Results

**Figure 1:**
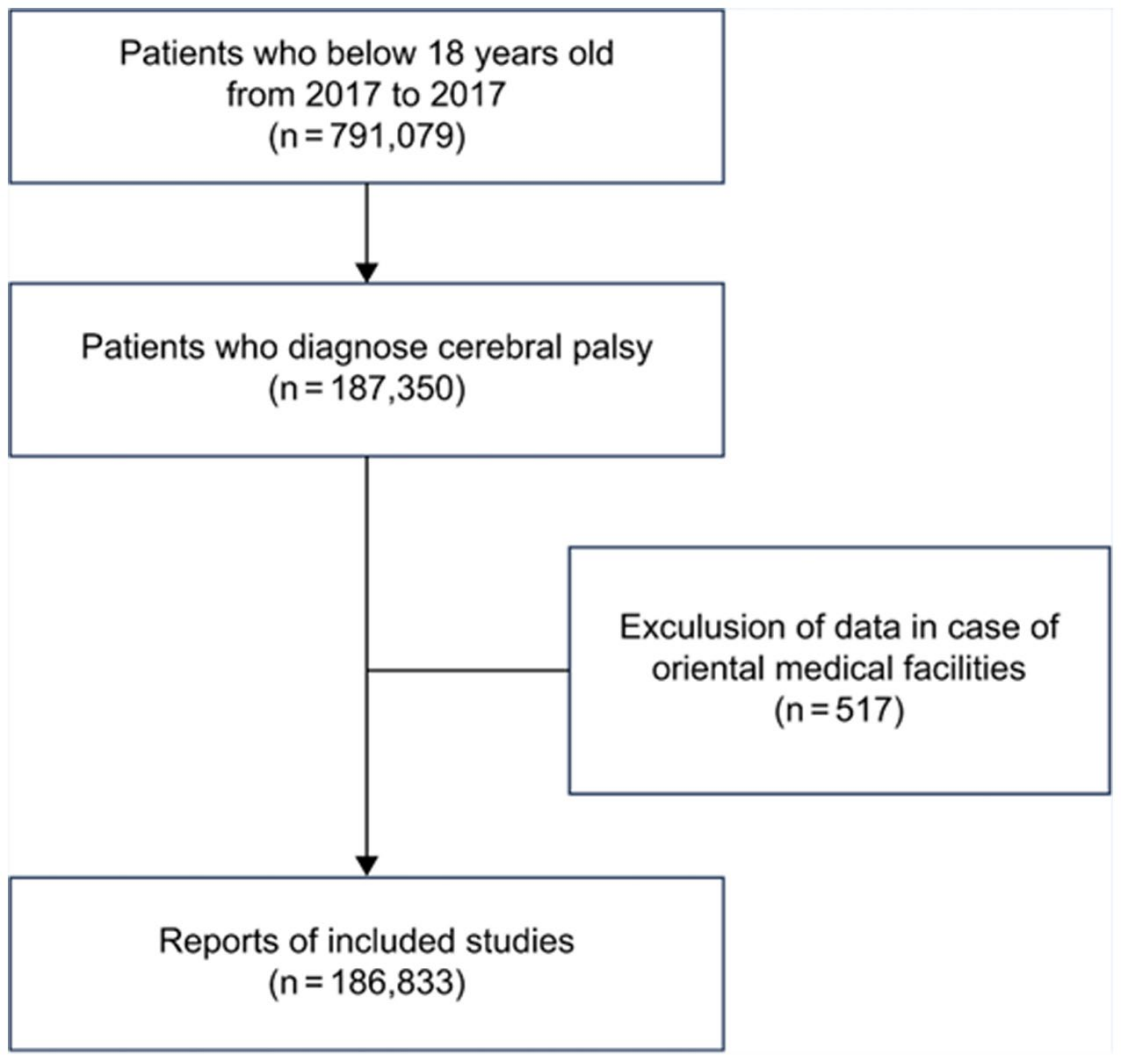
Flowchart of the study population.

Of the 791,079 individuals under 19 years of age, 187,350 were diagnosed with CP, and 517 individuals who received treatment at Oriental medicine hospitals were excluded. Consequently, 186,833 patients were included in the final analysis (Figure 1).

### 3.1 Characteristics of the study sample

Characteristics of participants are shown in Table 1. Data were categorized according to sex, age, type of medical institution, insurance type, and type of visit. The pre-pandemic and pandemic groups comprised 122,067 (65.3%) and 64,766 (34.7%) patients, respectively.

**Table 1:** Characteristics of participants.

		Before Covid 19						After Covid 19			
		2017		2018		2019		2020		2021	
		*n*	%	*n*	%	*n*	%	*n*	%	*n*	%
Sex	Males	24,570	58.0	23,878	58.2	22,451	58.0	19,528	58.1	17,889	57.4
	Females	17,777	42.0	17,165	41.8	16,226	42.0	14,093	41.9	13,256	42.6
Age (years)		7.38 ± 4.70		7.54 ± 4.68		7.67 ± 4.65		7.98 ± 4.58		8.08 ± 4.48	
Level of medical institution	Tertiary hospital	8,342	19.7	8,219	20.0	7,910	20.5	6,913	20.6	6,521	20.9
	General hospital	10,207	24.1	9,504	23.2	8,516	22.0	7,460	22.2	6,955	22.3
	Hospital	9,199	21.7	8,952	21.8	8,366	21.6	7,031	20.9	6,606	21.2
	Clinic	9,144	21.6	8,891	21.7	8,538	22.1	7,971	23.7	7,562	24.3
	Long-term care hospital	5,455	12.9	5,477	13.3	5,347	13.8	4,246	12.6	3,501	11.2
Insurance type	Health insurance	37,671	89.0	36,811	89.7	34,819	90.0	30,523	90.8	28,650	92.0
	Medical aid	4,676	11.0	4,232	10.3	3,858	10.0	3,098	9.2	2,495	8.0
Type of visit	Outpatient	31,376	74.1	29,973	73.0	28,367	73.3	25,275	75.2	23,229	74.6
	Inpatient	10,971	25.9	11,070	27.0	10,310	26.7	8,346	24.8	7,916	25.4
Total		42,347	100	41,043	100	38,677	100	33,621	100	31,145	100

### 3.2 Annual trends of rehabilitation

**Figure 2:**
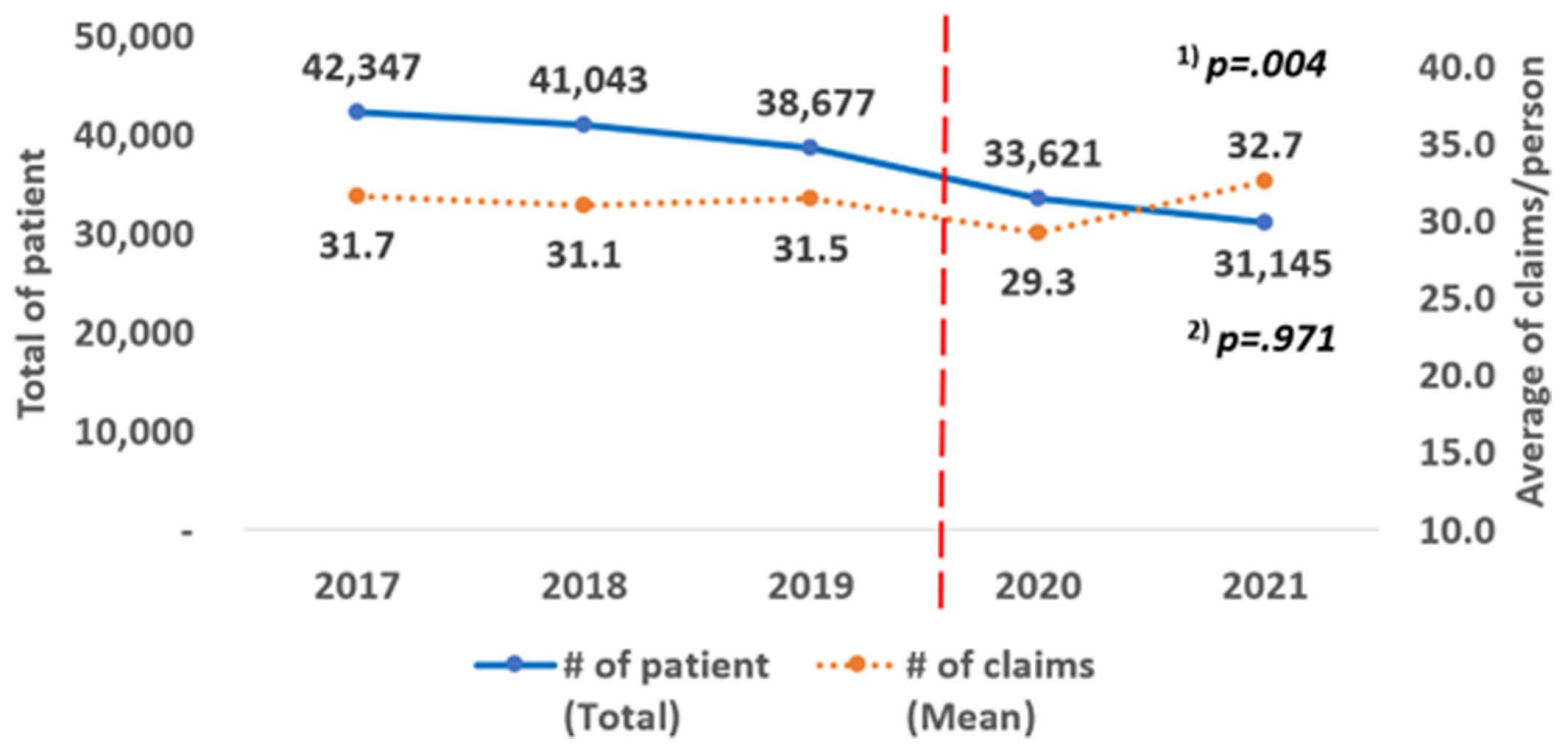
Annual change of the number of patients and claims. ^(1)^ P for trend according to the linear change in the total number of patients by year; ^(2)^ P for trend according to the linear change in the average number of claims/person by year.

The change in the number of patients who received rehabilitation therapy was significant (trend, *p* = 0.004), but the average number of claims per person did not show a statistically significant trend (trend, *p* = 0.971) over the 5 years. During the pre-pandemic period, the average number of claims per person was 31.4 (standard deviation, SD = 40.68). However, in the pandemic period, it decreased by an average of 0.5 cases to 30.91 (SD = 42.31), and this decrease was statistically significant (*p* = 0.013; Figure 2).

### 3.3 Depending on the claim codes for each rehabilitation program

**Figure 3:**
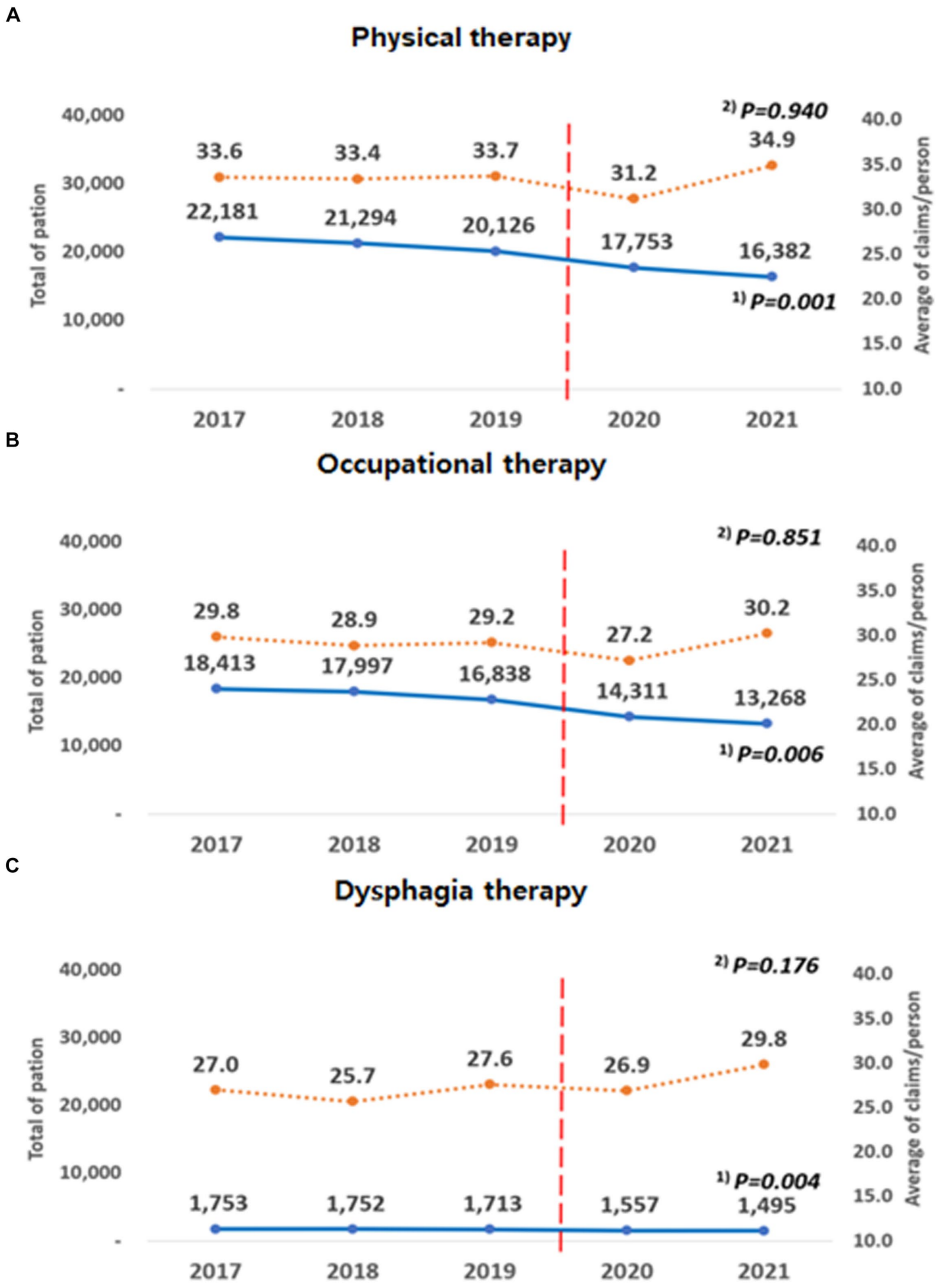
Annual changes according to the type of rehabilitation therapy. **(A)** (Physical therapy): MM105, MM301, MM302. **(B)** (Occupational therapy): MM112, MM113, MM114. **(C)** (Dysphagia therapy): MX141, MZ008. ^(1)^ P for trend according to the linear change in the total number of patients by year; ^(2)^ P for trend according to the linear change in the average number of claims/person by year.

The results of the analysis according to the type of rehabilitation therapy are shown in Figure 3. All three groups showed a decreasing trend in the number of patients over 5 years, and the results were statistically significant (PT group, trend *p* = 0.004, OT group, trend *p* = 0.006, DT group, trend p = 0.004). The average number of claims per person did not show a statistically significant trend (PT group: trend *p* = 0.940, OT group: trend *p* = 0.851, DT group: trend *p* = 0.176).

When the average number of claims per person before and during the pandemic was compared, a significant difference was observed between the PT and OT groups. In PT group, the average decreased from 33.56 (SD = 43.97) to 32.96 (SD = 45.90), a reduction of an average of 0.6 cases (*p* = 0.049). In OT group, the average decreased from 29.31 (SD = 36.67) to 29.66 (SD = 37.69), a reduction of an average of 0.7 cases (*p* = 0.019). In contrast, in the DT group, the average increased from 26.75 (SD = 36.14) to 28.34 (SD = 38.65), an increase of 1.5; however, this was not statistically significant (*p* = 0.065).

### 3.4 Based on the type of medical institutions

**Figure 4:**
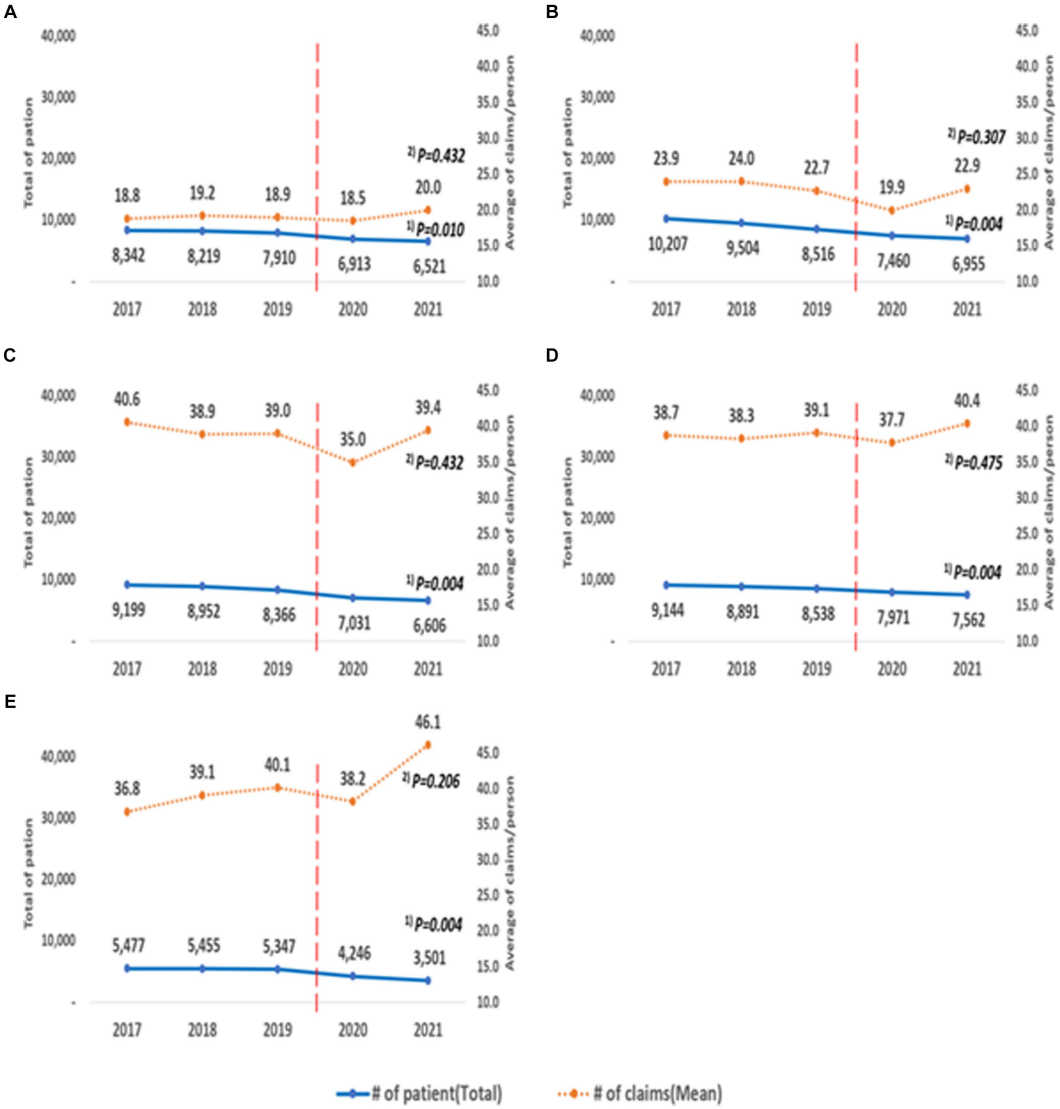
Annual changes according to the type of medical institutions. **(A)** Tertiary hospital, **(B)** General hospital, **(C)** Hospital, **(D)** Clinic, **(E)** Long term care hospital. ^(1)^ P for trend according to the linear change in the total number of patients by year; ^(2)^ P for trend according to the linear change in the average number of claims/person by year.

All institutions showed a statistically significant decreasing trend in the number of patients over the 5 years, and the results were statistically significant (tertiary hospitals: trend *p* = 0.010, general hospitals: trend *p* = 0.004, hospitals: trend *p* = 0.004, clinics: trend *p* = 0.004, long-term care hospitals: trend *p* = 0.004; Figure 4). The average number of claims per person did not show a statistically significant trend (tertiary hospitals: trend *p* = 0.432; general hospitals: trend *p* = 0.307; hospitals: trend *p* = 0.432; clinics: trend *p* = 0.475; long-term care hospitals: trend *p* = 0.206).

When comparing the average number of claims per person before and during the pandemic, statistically significant changes were observed in general hospitals, hospitals, and long-term care hospitals. The general hospital average decreased from 23.57 (SD = 25.03) to 21.39 (SD = 23.40), a reduction of an average of 2.2 cases (*p* < 0.001). The hospital average decreased from 39.51 (SD = 51.44) to 37.12 (SD = 48.60), a reduction of 2.4 cases (*p* < 0.001). Conversely, the long-term care hospital average increased from 38.64 (SD = 47.19 to 41.78 SD = 55.02), an increase of an average of 3.1 cases (*p* < 0.001).

For tertiary hospitals, the average increased from 18.96 (SD = 17.81) to 19.19 (SD = 20.54), an increase of an average of 0.2 cases, but this was not statistically significant (*p* = 0.254). For clinics, the average increased from 38.71 (SD = 47.76) to 39.02 (SD = 51.45), an increase of an average of 0.3 cases, but this was also not statistically significant (*p* = 0.546).

### 3.5 Hospitalization and outpatient visits

**Figure 5:**
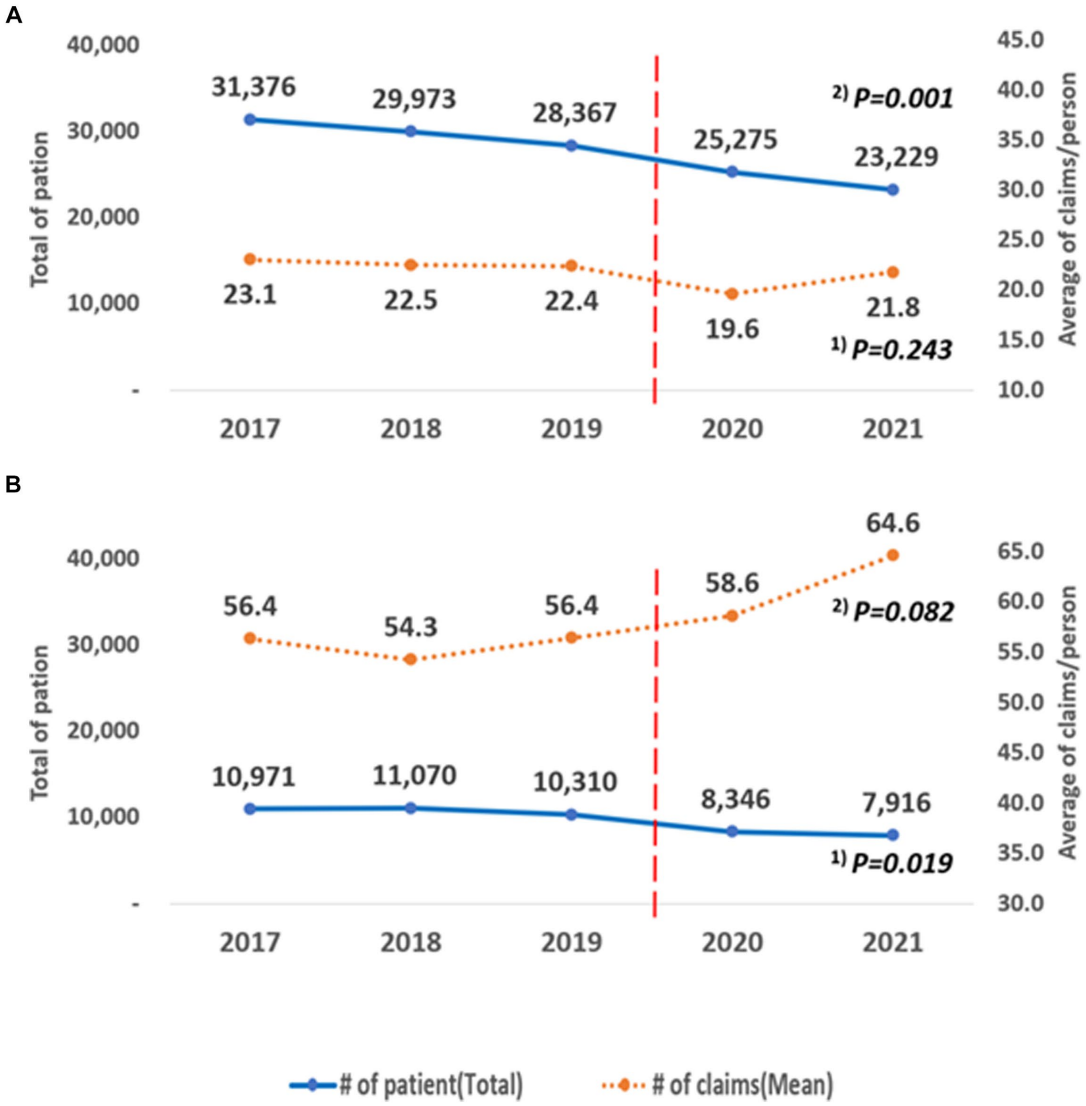
Annual changes according to the type of inpatients and outpatients. **(A)** Outpatient, **(B)** Inpatient. ^(1)^ P for trend according to the linear change in the total number of patients by year; ^(2)^ P for trend according to the linear change in the average number of claims/person by year.

The results of hospitalization and outpatient visits are shown in Figure 5. Over the 5 years, the number of patients showed a decreasing trend, and the results were statistically significant (patients: trend *p* = 0.001, Outpatients: trend *p* = 0.019). The average number of claims per person did not show a statistically significant trend (patients: *p* = 0.243, outpatients: *p* = 0.082). When comparing the average number of claims per person before and during the COVID-19 pandemic, there were statistically significant differences between outpatients and inpatients. For outpatients, the average decreased from 22.67 (SD = 23.90) to 20.65 (SD = 22.59), a reduction of 2.0 cases (*p* < 0.001). For inpatients, the average increased from 55.66 (SD = 62.13) to 61.53 (SD = 66.01), an increase of an average of 5.9 cases (*p* < 0.001).

### 3.6 Depending on health insurance types

**Figure 6:**
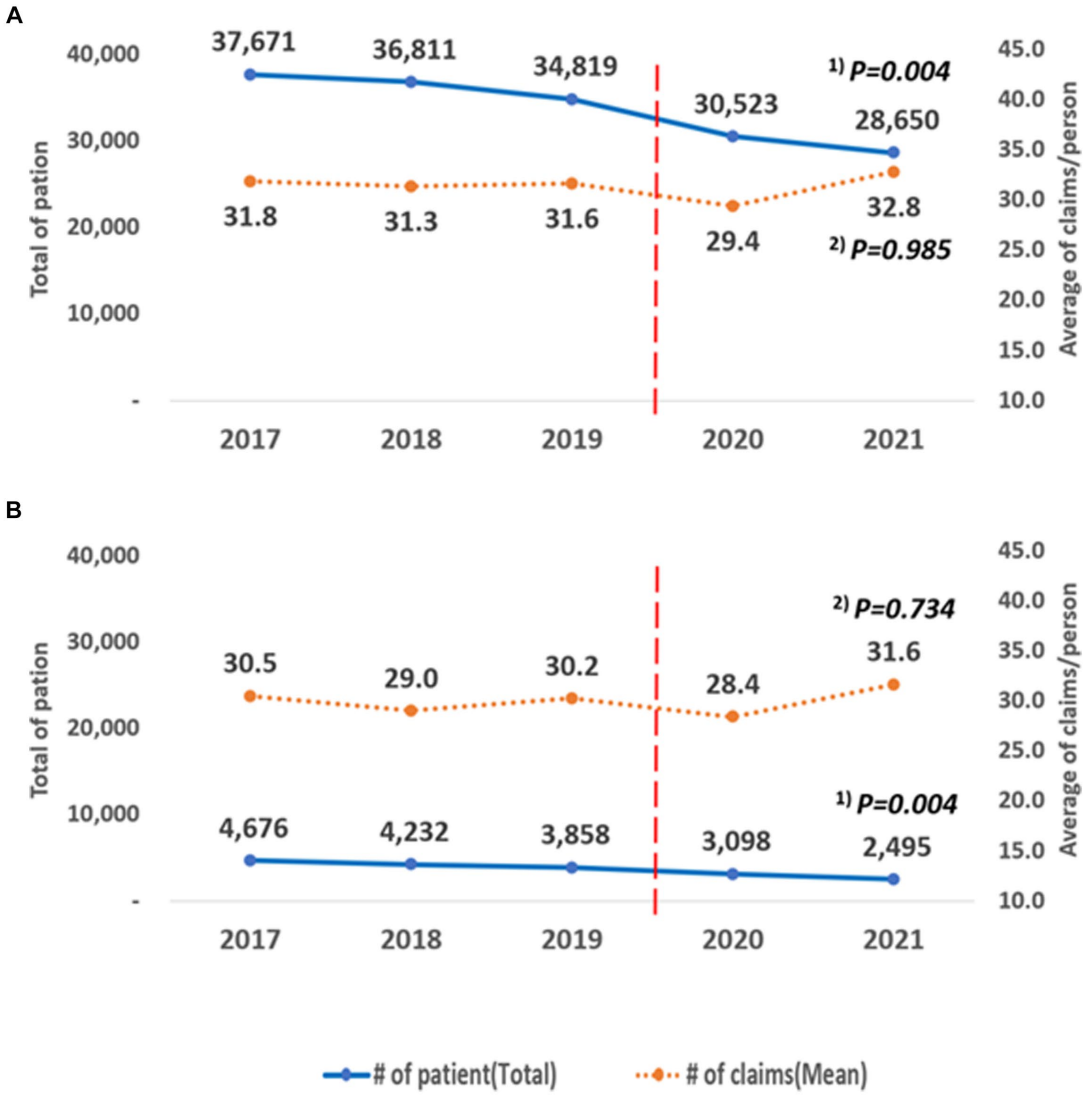
Annual changes according to the type of insurance. **(A)** Health insurance, **(B)** Medical aid. ^(1)^ P for trend according to the linear change in the total number of patients by year; ^(2)^ P for trend according to the linear change in the average number of claims/person by year.

Over the 5 years, the number of patients showed a decreasing trend, and the results were statistically significant (National health insurance: trend *p* = 0.004, medical aid: trend p = 0.004; Figure 6). The average number of claims per person did not show a statistically significant trend (health insurance: trend *p* = 0.985, medical aid: trend *p* = 0.734). When comparing the average number of claims before and during the pandemic, there was a statistically significant difference in health insurance coverage. For those covered by health insurance, the average decreased from 31.59 (SD = 41.09) to 31.01 (SD = 42.37), a reduction of an average of 0.5 cases (*p* = 0.007). For those covered by medical aid, the average decreased from 29.91 (SD = 36.92) to 29.82 (SD = 41.63), a reduction of 0.08 cases, but this was not statistically significant (*p* = 0.898).

## 4 Discussion

In CP, rehabilitation therapy plays a significant role in physical function, activities of daily living, and social participation. We analyzed the impact of the COVID-19 pandemic on children and adolescents with CP who were undergoing rehabilitation therapy in Korea using national surveillance data. Our findings highlight the significant influence of the pandemic on rehabilitation processes.

Discontinuation of rehabilitation can lead to multiple complications, such as skeletal deformities, impaired motor abilities, swallowing difficulties, and respiratory function challenges. Moreover, children with neurological disorders may experience significant deterioration in motor capabilities (10). Bhaskar et al. (11) reported that, during the COVID pandemic, owing to the lack of rehabilitation, orthotic support, and lockdown restrictions, 53.5% of children experienced a deterioration in physical function, and 33.7% experienced a worsening of deformities. Cankurtaran et al. (12) reported that 53% of children with CP had interruptions in their routine PT sessions, with breaks lasting an average of 3 months.

### 4.1 Annual trends of rehabilitation

Our results showed that the total number of patients consistently decreased from 2017 to 2021. However, no significant changes were observed in the average number of claims per person. Notably, there was a decline after the onset of the pandemic in 2020; however, it rebounded with an increase in 2021. In the early stages of the pandemic, people refrained from visiting medical institutions because of the government’s lockdown policies and fear of contracting COVID-19 (13). Children with disabilities face challenges in wearing masks owing to issues such as respiratory problems, difficulty with cooperation, and masks becoming wet with saliva. Thus, wearing masks may have made it more difficult for them to participate in rehabilitation programs. However, with the easing of social distancing measures and the high vaccination rate, health service utilization gradually increased, rebounding to pre-pandemic levels by December 2022 (14).

### 4.2 Depending on the claim codes for each rehabilitation program

Claim codes were categorized into PT, OT, and DT groups. Bertamino et al. (6) reported that, during the pandemic, 26.7 and 41.7% of children with pediatric and perinatal stroke undergoing physiotherapy and OT, respectively, discontinued their treatment. Furthermore, a French study indicated that only 48, 27, 32, 31, and 13% of children continued their sessions in physiotherapy, OT, speech therapy, psychomotor therapy, and orthopedics, respectively (15). During the pandemic, we expected that DT would significantly decline due to the potential direct exposure to respiratory droplets from patients. However, while the average claims per individual decreased for the PT and OT groups before and during COVID-19, DT increased by an average of 1.5. Miles et al. (16) recommended continuing dysphagia assessment and treatment procedures during the COVID-19 pandemic with infection prevention measures.

### 4.3 Depending on the type of medical institutions

Depending on the medical institution, the average number of claims per person during the pandemic decreased significantly in general hospitals and hospitals. By contrast, long-term care hospitals experienced an average increase of 3.1 cases. In response to the spread of COVID-19, the government has designated almost all public, general, and long-term care hospitals as dedicated COVID-19 facilities. Consequently, the number of beds for patients receiving rehabilitation treatment decreased. During the COVID-19 pandemic, public hospitals providing general healthcare services saw a significant reduction in healthcare utilization; however, no significant changes were reported in healthcare utilization at specialty, disease-centered, and long-term care hospitals (17). However, there were no changes in specialized target/disease-centered institutions or older adult hospitals. Although a decline in the number of patients due to COVID-19 was common across all medical institutions, the extent varied depending on the size of the medical facility (18). According to a study using NHIS data on the impact of COVID-19 on healthcare utilization, the pandemic had a minimal effect on outpatient and inpatient services at higher level hospitals, and the number of outpatient visits and inpatients at long-term care facilities decreased (14).

### 4.4 Depending on hospitalization and outpatient visits

During the pandemic, the average number of claims per outpatient decreased by 2.0 cases, while inpatients experienced an average increase of 5.9 cases. In 2020, medical usage dropped by 14.5% for both inpatients and outpatients compared with the previous year (19). Similar trends were reported worldwide (20, 21). The study found outpatient rehabilitation claims decreased in 2020 but rose in 2021, while inpatient claims consistently increased. Disruption of outpatient services during the pandemic likely led to more intensive inpatient rehabilitation. Despite fewer inpatients, the treatment frequency and intensity for those requiring rehabilitation increased, with resumed and intensified sessions as protocols evolved.

### 4.5 Depending on health insurance types

Insurance data showed a 0.5 case decrease in average claims per person for national health insurance patients, with no significant change for medical aid patients. A study on income-based healthcare utilization using NHIS data reported that the higher economic group experienced greater outpatient service utilization reductions than pre-COVID-19 levels. The smallest reduction was observed among Medicaid recipients and the poorest patient groups, specifically those in the 0–20th income percentile. In rehabilitation therapy, the decrease in claims among individuals receiving medical aid was likely less prominent.

The COVID-19 pandemic abruptly challenged the pediatric rehabilitation field and the broader healthcare system, leaving them unprepared to support children and families effectively (6). During the pandemic, 67.7% of people with developmental disabilities cited infection risk as their primary concern when accessing hospital services, followed by difficulties with mask-wearing and COVID-19-related hospital restrictions (22). However, the reduction or cessation of hospital-based rehabilitation treatments was not solely negative. Pizzighello et al. (23) observed a temporary dip in children’s rehabilitation, with few parents finding alternatives, yet increased family time was seen positively. Similarly, Cankurtaran et al. (12) found that parents and adolescents with disabilities valued the additional family time. Ben-Pazi et al. (24) suggested that, as children with CP stay at home, their participation in the household increases, and the use of telemedicine can help narrow the gap with the general population. Bertamino et al. (6) reported that telerehabilitation, with sustained or increased home stimulation by parents, led to 82.1% perceiving their child’s condition as stable or improved while keeping up therapy contacts. Parents need to utilize resources to navigate the pandemic’s health impacts with government support. Studies have been undertaken on the necessity and feasibility of telerehabilitation, but an integrated discussion is notably absent. Serious deliberations are required concerning the suitable indications, methodologies, and protocols for incorporating it within the established healthcare system.

### 4.6 Limitations and strength

This study has limitations. Firstly, it relied on claims data from medical institutions, which means it could not capture rehabilitation trends in non-medical settings during the pandemic. Thus, it may not completely represent the treatment experiences of all patients. Secondly, the study only focused on the years 2020–2021, representing the initial phase of the pandemic without analyzing annual changes. Further research is needed to assess the long-term effects of COVID-19. Despite these limitations, the study is significant because it offers a comprehensive overview of the impact of the pandemic on rehabilitation services for children and adolescents with cerebral palsy.

## 5 Conclusion

During the COVID-19 pandemic, rehabilitation visits for pediatric cerebral palsy patients dropped, with claims varying by treatment and institution. We assessed the pandemic’s impact on these services, providing data to guide improved policies and care practices. This study clarifies the adaptability of rehabilitation therapy during such crises.

## Data availability statement

Publicly available datasets were analyzed in this study. This data can be at: https://opendata.hira.or.kr/or/orb/bigInfo.do#.

## Ethics statement

The study protocol was approved by the Institutional Review Board of HIRA (IRB No. 2022–035-001). Informed consent was waived because of the retrospective nature of the study.

## Author contributions

JS: Formal analysis, Validation, Visualization, Writing – original draft, Writing – review & editing. MH: Investigation, Project administration, Supervision, Writing – original draft, Writing – review & editing. JP: Validation, Writing – review & editing. YL: Supervision, Visualization, Writing – review & editing.
